# Indigenous knowledge of zootherapeutic use among the Biate tribe of Dima Hasao District, Assam, Northeastern India

**DOI:** 10.1186/1746-4269-9-56

**Published:** 2013-08-12

**Authors:** Albert Lalduhawma Sajem Betlu

**Affiliations:** 1 North Cachar Hills Community Resource Management Society (NCHCRMS-NERCoRMP), Sarkari Bagan, Haflong, Dima Hasao, India

**Keywords:** Ethnozoology, Zootherapy, Biate, Dima Hasao, North Cachar Hills

## Abstract

**Background:**

The present study addresses the use of zootherapy in the traditional healthcare system of the Biate tribe of Dima Hasao district, Assam, India. It sought to identify the different species used for zootherapeutic use with the detailed methods of usages to create awareness and contribute to the conservation and sustainable utilization of the resources.

**Method:**

15 Biate villages within the district of Dima Hasao were surveyed through semi-structured questionnaires and informal interviews. Detailed information on the uses of each animal was recorded. Species were identified using standard literature. Fidelity level (FL) was calculated to demonstrate the percentage of respondents claiming the use of a certain animal for the same major purposes.

**Result:**

The study documents 34 species for the treatment of about 34 different ailments. The largest number of species reported was mammals with17 species. Maximum number of species has been reported for the treatment of diabetes and its high fidelity levels warrants in-depth studies to establish its pharmacological activity. The usages documented herein are unique to the Biate tribe. Very often, these animals are hunted and sold openly at the local markets in the lure of quick money. A 300 gm live *Gekko gecko* may fetch a sum of 2,50,000 Indian Rupees (INR), and smoked meat of *Hoolock hoolock* cost approximately 250–300 INR per kg. Animals are also hunted for its hide. The unrestricted hunting of species like *Capricornis sumatraensis* has almost wiped out the population within the district. Some species are also reared as pets while some are used for display as a sign of expertise in hunting. The present study has documented the usage of at least 15 animals listed in the IUCN Red List.

**Conclusion:**

The study illustrates the in-depth knowledge of the Biate tribe on zootherapy. Systematic investigation to identify the active ingredient may lead to the development of new drugs, which would prompt protection of these valuable resources.

## Background

Animal parts have been used as a source of medicine in the traditional healthcare system since ancient times, and have played a significant role in the healing practices. In the modern societies, zootherapy constitutes an important alternative among many other known therapies practiced worldwide [1]. The World Health Organization estimates that as many as six billon people rely primarily on medicines of animal and plant origin [2]. Although plant and plant derived materials are more commonly used in traditional medical systems than animal derived products, the latter also constitute an important element in most of the traditional healthcare systems of the world. As for example, Traditional Chinese Medicine (TCM) recorded more than 1,500 animal species to be of medicinal use [3]. A list of 60 different species of insects used to treat a wide range of disabilities and illnesses in Japan has been published [4] and 24 animal species were identified, whose by-products were used therapeutically by the Tamang people of Nepal [5]. However, in spite of such worldwide prevalence, research on medicinal animals has often been neglected in comparison to medicinal plants [6]. Pieroni et al. [7], for example, has pointed out that studies on drugs of animal origin are still rare in the scientific literature.

India is known for its age-old heritage of traditional medicine. It is a goldmine of well-recorded and traditionally practiced knowledge of herbal medicine such as the Ayurveda, Siddha and Unani medical system. India officially recognizes over 3,000 plants for their medicinal value and estimated that over 6,000 plants are in use in traditional, folk and herbal medicine, representing about 75% of the medicinal needs of the third world countries [8]. Nonetheless, as far as zootherapeutic uses is concerned, very few researches has been done so far, especially in the northeastern states of India. Northeastern India harbors over 130 major tribal communities. Most tribal communities still largely depend on their traditional system of medicine. Because of their scattered and far-flung settlements, and problems arising due to transportation and communication, traditional medicine has remained as the most affordable and easily accessible source of treatment [9]. Although some of the traditional medicines have already been documented, tried, tested and even incorporated in the modern systems of medicine, much larger number of these folk medicines is still undocumented and remains endemic to certain pockets of the area. Each tribe have their own unique way of traditional healthcare system that has been handed down from generation to generation orally, but with the steady development of rural areas coupled with the lure for a more profitable job, the interest of the younger generations in this tradition is declining. The traditional ecological knowledge including the healing practice is gradually being corroded from their culture. Therefore, ethnobiologist in this region have a greater responsibility not only in inventorizing the traditionally used biological resources but also in conserving and revitalizing the traditional beliefs, so that these age-old cultures are not lost [10].

Because of their rich traditional knowledge, different tribal communities of northeastern India have been a subject of several intense and eye-opening ethnozoological studies. Solanki and Chutia reported 11 species used by the Monpa tribe of Arunachal Pradesh [11], Lalramnghinglova has reported the usage of 56 species from the state of Mizoram [12], Chakravati et al. reported the usage of 36 species from Arunachal Pradesh [13], Jamir et el. reported 26 species from Nagaland [14] while Kalita reported the usage of 4 species from Assam [15]. Kakati and Duolo reported 23 species from Nagaland [16] while Kakati reported 25 species used by the Ao Naga [17]. The district i.e. Dima Hasao of Assam, formerly North Cachar Hills district, has also been subjected to several ethnobotanical studies [18-26]. Nevertheless, till date, no comprehensive report on the ethnozoological wealth and indigenous knowledge of zootherapeutic use is available from the district. The present study area remains to be explored in this aspect. It is on this backdrop that the present research work has been taken-up for the first time to document the indigenous zootherapeutic knowledge of the Biate tribe of Dima Hasao district of Assam, northeastern India.

### The study area

**Figure 1:**
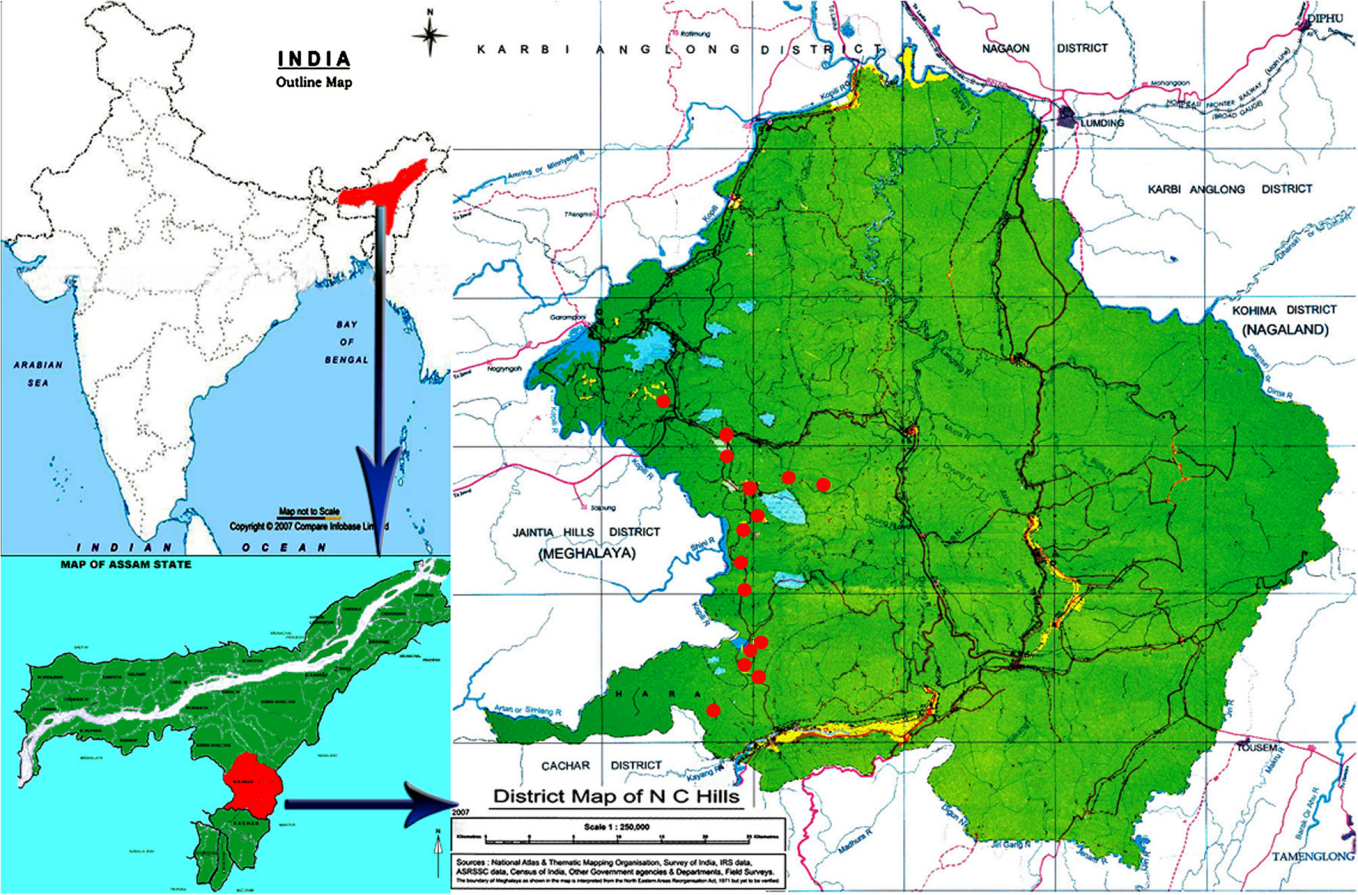
Map showing the location of the study area.

Dima Hasao district, covering an area of 4890 km^2^ (92°37^/^ E – 93°17^/^E longitude and 25°3^/^ N – 25°27^/^ N latitudes) is a place of immense interest and potential for ethnobotanist, ethnozoologist as well as anthropologist (Figure 1). More than 12 ethnic tribes namely Dimasa, Zeme, Biate, Jaintia, Hrangkhol, Hmar, Kuki, Vaiphei, Khelma, Rongmei, Lushai, Karbi etc., live harmoniously together within the district with several non tribal groups like the Assamese, Bengali, Nepali, etc. who are mostly government employees, traders, living in urban and semi-urban areas. It has a total population of 213,529 and the density of population is 44 individuals per square kilometers which is the lowest in the state of Assam according to the census of India, 2011 [27]. Most of the villages are situated far from modern conveniences and inaccessible by road or rail. Thus, the tribal villagers have deep faith in their traditional healthcare system and in most cases prefer them to the modern system of medicine.

### The Biate tribe

**Figure 2:**
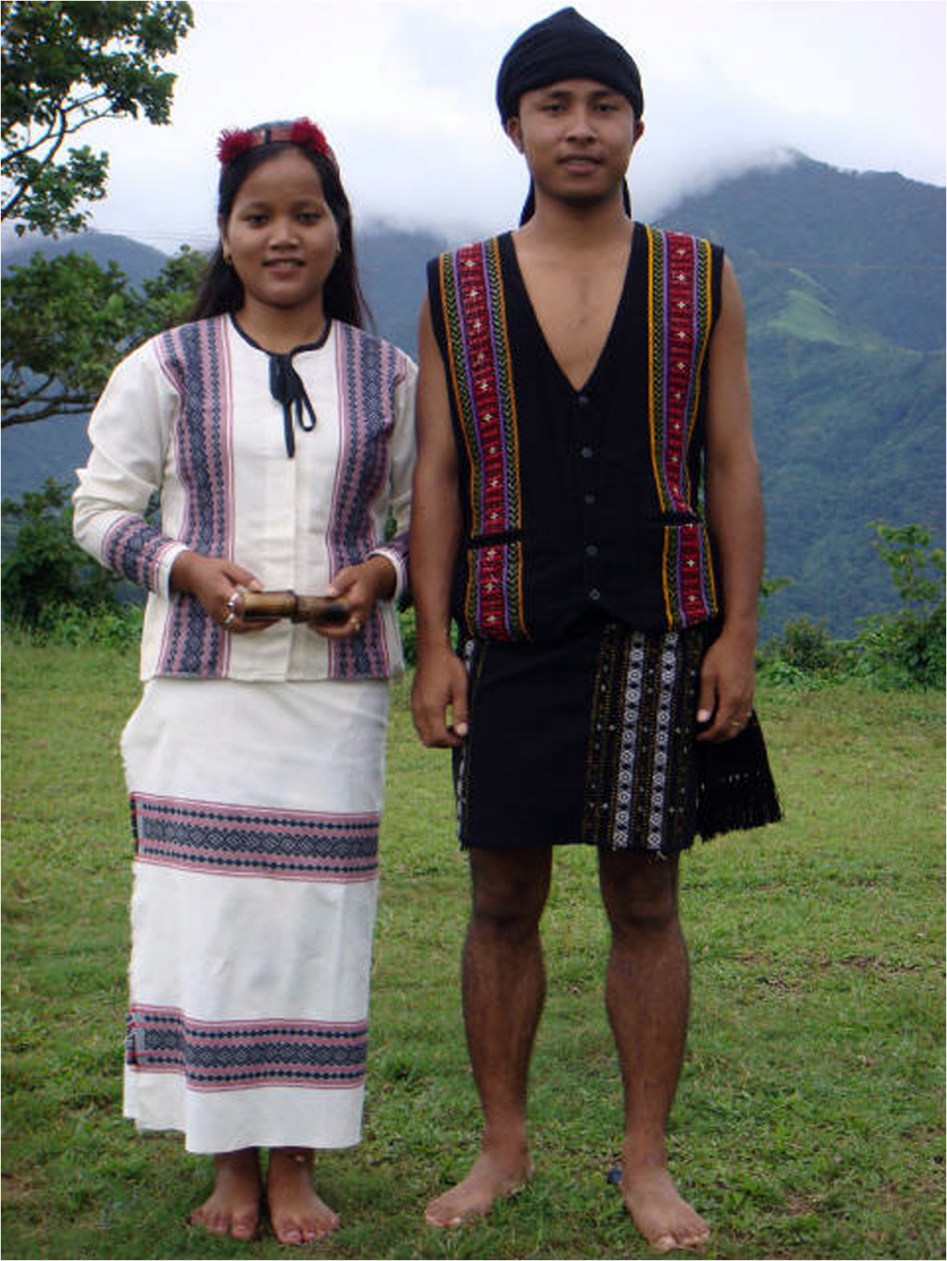
A Biate couple in traditional attire.

The Biate tribe (Figure 2) belonging to one of the branches of Mongoloid stock of race is among the prominent inhabitants of the district occupying the Kharthong constituency spreading into the other side of the Kopili River in the Saipung constituency of Jaintia Hills of Meghalaya [28]. Shifting cultivation is the traditional means of agriculture practiced by the villagers. Besides these, they raise livestock such as cows, goats, pigs, chicken and also grow a variety of both wild and cultivated plants in their residential compounds.

In olden days, the Biate people believe in naturalism, animism and animalism. Besides them, they believed that there is the high God called *Chung Pathian* which means Heavenly Father. When any disease or fatal epidemic comes through the village, the priest (*Puithiam or Thiampu*) performs the rituals calling upon the *Chung Pathian* to protect the village from the epidemic and the entire village is out of bounds to outsiders for five days or more [29]. However, today, with the advent of Christianity and majority of them being Christians, most of these rituals have been discarded and with it scores of traditional knowledge including ethnomedicine has been forgotten.

### Methodology

The present study is based on a carefully planned, field survey on 15 Biate villages within the district of Dima Hasao. Surveys were carried out from November 2010 to March 2013 with a minimum of three visits to each village, to gather information on a maximum number of species. Surveys were conducted through semi-structured questionnaires and in cases where the respondents were uncomfortable with the questionnaires, discussion and informal interviews were employed, and in the process information on different zootherapeutic uses were noted and documented [30,31]. A total of 300 individuals were interviewed. The age of the respondents ranges between 27 years to 78 years and the number of male respondents was higher (67%) as compared to the female respondents (33%). The respondents were selected randomly and Prior Informed Consent (PIC) was obtained for all interviews conducted. Information on the uses of each animal for disease treated, method of preparation, use and dosage, side effects, spiritual aspects linked to its use, parts used as medicine, local name, whether dried or fresh, whether rare or abundant, changes in abundance of the animal for the last 10 years, food value with market rate, efficacy of the remedies and how this knowledge was acquired by the respondents themselves were recorded. The scientific name and species of animals were identified by using relevant and standard literature.

### Data analysis

For the data analysis, Fidelity Level (FL) calculated that demonstrates the percentage of respondents claiming the use of a certain animal for the same major purpose, was calculated for the most frequently reported diseases or ailments as,

FL (%) = *N*p × 100/*N*.

Where *N*p is the number of respondents that claim a use of a species to treat a particular disease, and *N* is the number of respondents that use the animals as a medicine to treat any given disease [31].

The range of fidelity level (FL) is from 1% to 100%. high use value (close to 100%) show that this particular animal species are used by large number of people while a low value show that the respondents disagree on that species to be used in the treatment of ailments.

## Results and discussion

**Figure 3:**
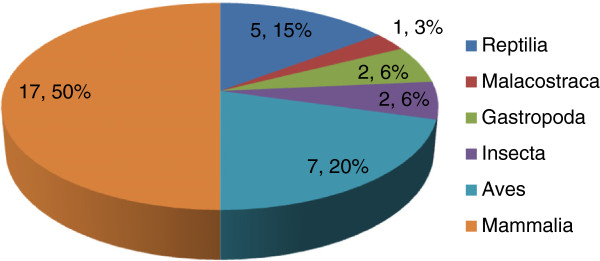
Proportional representation of total number of species and the percentage of contribution amongst each Class.

The present study documents 34 species used for the treatment of different ailments. Table 1 summarizes the scientific names of the medicinally used species, their vernacular names, the part(s) of the species used, the diseases or ailments the animal derived medicines thought to be effective for, and the ways the treatments are carried out. Table 2 summarizes the present conservation status of the species mentioned in Table 1 as zootherapeutically important. These species are distributed among at least 31 zoological families. The taxonomic Class with the largest number of species were Mammalia (17 species), followed by Aves (7 species) and Reptilia with five species (Figure 3). In similar studies carried out around the world, mammals and birds also recorded the highest use as part of local folk medicines [32-35]. Surveys other than our own research from northeastern India indicates the same [14,16,17]. Other Class mentioned by the interviewees belonged to Gastropoda (2 species), Insecta (2 species) and Malacostraca (1 species). Different researchers from India have already contributed to the knowledge of 351 ethno medicinal uses of animal and its parts (Table 3) [11-17,36-44]. The present study, with the usage of 34 species recorded for the first time from the district is an addition to the above figure.

**Table 1:** Medicinal uses of animals and animal parts in traditional therapy by the Biate tribe of Dima Hasao district

**Scientific Name/Family**	**English name**	**Part used**	**Dried/Fresh**	**Disease treated**	**Method of preparation, use and dosage of medicine**	**Used Singly/combination with others**	**Side effects reported**	**Fidelity level (%)**
**Class: Reptilia**								
*Ophiophagus hannah* (Cant. 1836) /Elapidae	King Cobra	Gall bladder	Dried	Snake bite	Eaten/swallowed whole. 1 whole piece once.	Singly	None	33
*Python molurus* (Linn., 1758) /Pythonidae	Python	Gall bladder	Dried	Diabetes	Eaten/swallowed whole. 1 whole piece once every year.	Singly	None	56
		Gall bladder	Dried	Snake bite	Eaten/swallowed whole. 1 whole piece once.	Singly	None	38
		Flesh	Dried or fresh	Seizure	Eaten cooked with vegetables. No particular dosage.	Singly or in combination with vegetables	None	90
		Fat	Fermented	Sprain	Applied locally	Singly	None	38
		Fat	Fermented	Piles	Applied locally in piles	Singly	None	82
*Varanus bengalensis* (Daudin, 1802) /Varanidae	Monitor Lizard	Fat	Fermented	Burns	Applied locally	Singly	None	61
		Flesh	Dried	Jaundice	Cooked and eaten. No particular dosage.	Singly	None	54
*Gekko gecko* (Linn. 1758) /Gekkonidae	Tokay Gecko	Flesh	Dried	Impotency	Cooked and eaten. No particular dosage.	Singly	None	38
*Melanochelys trijuga* (Schw.1812) /Geoemydidae	Indian Pond Terrapin	Flesh	Fresh/Dried	Allergy	Cooked and eaten. No particular dosage.	Singly	None	24
**Class: Malacostraca**								
*Paratelphusa* sp. (Alcok, 1919) /Paratelphisidae	Fresh water crab	Whole	Fresh	Jaundice	Crabs (especially small ones) are crushed to pulp. The juice extracted is mixed with a little water and boiled till it becomes half of the quantity and then taken. It is often cooked along with banana flower. No particular dosage.	Singly	None	96
**Class: Gastropoda**								
*Cryptozona* sp (Moerch, 1872) /Ariophantidae	Snail	Whole	Fresh	Rheumatism and Sciatica	Inserted inside a banana for ease of ingestion and swallowed whole. 1 every day for 1 week.	Singly or in combination with edibles	None	36
*Lymnaea* sp. (Lam. 1822) /Lymnaeidae	Water snail	Flesh	Fresh	Jaundice	Cooked and eaten. No particular dosage.	Singly or in combination with vegetables	None	73
**Class: Insect**								
*Cimex lectularius* (Linn.1758) /Cimicidae	Bed bugs	Whole	Fresh	Malaria	3 bedbugs are swallowed whole, daily for 1 week. It is often inserted in a banana for ease of ingestion.	Singly	None	27
*Periplanata americana* (Linn. 1758) /Blattidae	Cockroach	Whole	Dried	Tuberculosis	Crushed and eaten. 3 every week for 1 month.	Singly or in combination with edibles	None	2
**Class: Aves**								
*Gallus gallus* (Linn. 1758) /Phasianidae	Jungle fowl	Fat	Fermented	Burns	Applied locally.	Singly	None	83
*Corvus macrorhynchos* (Wagler, 1827) /Corvidae	Jungle crow	Flesh	Dried or fresh	Health tonic for the aged	Cooked and eaten. No particular dosage.	Singly or with vegetables	None	82
*Rhyticeros undulatus* (Shaw, 1811) /Bucerotidae	Wreathed Hornbill	Fat	Fermented	Burns	Applied locally.	Singly	None	45
*Passer domesticus* (Linn. 1758) /Passeridae	House Sparrow	Brain	Fresh	Impotency	Cooked and eaten. No particular dosage.	Singly or with vegetables	None	27
*Myophonus caeruleus* (Scopoli, 1786) /Turdidae	Blue whistling Thrush	Flesh	Dried	Meat allergy	Cooked and eaten. No particular dosage.	Singly or with vegetables	None	37
*Buceros bicornis* (Linn. 1758) /Bucerotidae	Great Hornbill	Fat	Fermented	Arthritis	Applied locally.	Singly	None	60
*Upupa epops* (Linn. 1758) /Upupidae	Hoopoe	Flesh	Dried	Gall bladder stone	Cooked and eaten. No particular dosage.	Cooked with *Marsdenia sp*	None	75
**Class: Mammalia**								
*Manis crassicaudata* (E. Geof. 1803) /Manidae	Indian Pangolin	Scales	Dried	Hook worm	1 scale, crushed to powder and taken with water.	Singly	None	87
*Canis aureus* (Linn. 1758) /Canidae	Golden Jackal	Flesh	Dried	Tuberculosis	Cooked and eaten. No particular dosage.	Singly or with vegetables	None	48
		Flesh	Fresh	Joint pain	Cooked and eaten. No particular dosage.	Singly or with vegetables	None	44
		Gall bladder	Dried	Malaria	Swallowed whole. 1 whole piece once.	Singly	None	36
*Muntiacus muntjak* (Zimm. 1780) /Cervidae	Barking Deer	Flesh	Dried or fresh	Easy conception	Cooked and eaten. No particular dosage.	Singly or with vegetables	None	57
*Eonycteris spelaea* (Dobson, 1871) /Pteropodidae	Bat	Flesh	Dried	Eneuresis	Cooked and eaten. No dosage	Singly	None	81
*Melursus ursinus* (Shaw, 1791) /Ursidae	Sloth Bear	Gall bladder	Dried	Diabetes	Swallowed whole atleast twice in a year	Singly	None	78
		Gall bladder	Dried	Easy delivery of a child	Swallowed whole before delivery. 1 whole piece once.	Singly	Decreases lactation which becomes normal again after 3–6 days.	93
*Ursus thibetanus* (Cuvier, 1823) /Ursidae	Himalayan Black Bear	Gall bladder	Dried	Diabetes	Swallowed whole atleast twice in a year.	Singly	None	82
		Gall bladder	Dried	Easy delivery of a child	Swallowed whole before delivery. 1 whole piece once.	Singly	Decreases lactation which becomes normal again after 3–6 days.	89
*Hoolock hoolock* (Harlan, 1834) /Hylobatidae	Hoolock Gibbon	Gall bladder	Dried	Diabetes	Swallowed whole. 1 whole piece once.	Singly	None	87
		Flesh	Dried	Tonic for pregnant women	It is cooked with vegetables and taken. No dosage	In combination with different vegetables	None	80
		Brain	Fresh	Tonic for pregnant women	Cooked and eaten. Believed to be an excellent health tonic especially for a pregnant women and her unborn child. No dosage	Singly	None	68
		Bone	Dried	Rheumatism	A piece of bone is usually tied around the foot, leg or waist	Singly	None	97
		Skull bone	Dried	Dizziness	Piece of skull bone is tied to the head.	Singly	None	3
		Hand	Dried	Hernia	Smoked dried hand is used to rub down the affected area.	Singly	None	16
*Capricornis sumatraensis* (Bechstein, 1799) /Bovidae	Mainland Serow	Front foot	Dried	Joint pains, rheumatism	The affected area is usually hammered gently every day	Singly	None	74
		Urine	Fresh	Diabetes	~ 5 ml once in a month.	Singly	None	24
*Nycticebus coucang* (Boddaert, 1785) /Lorisidae	Slow Loris	Fur	Dried	Cuts & wounds and bed sore	Applied locally	Singly	None	31
*Canis lupus familiaris* (Linn. 1758) /Canidae	Dog	Blood	Fresh warm	Chronic malaria	~ 100 ml of fresh blood is drunk while still warm	Singly	None	58
		Blood	Fresh warm	Epilepsy	~ 50 ml fresh blood mixed with alcohol (preferably local rice beer) is taken once.	Singly	Slight dizziness and nausea	10
		Flesh	Dried or fresh	Epilepsy	Cooked and eaten. No dosage	In combination with different vegetables	None	38
*Macaca assamensis* (McClelland, 1840) /Cercopithecidae	The Assamese Macaque	Flesh	Dried	Tonic for pregnant women	Cooked and eaten. No dosage	In combination with different vegetables	None	61
		Brain	Fresh	Tonic for pregnant women	Cooked and eaten. Believed to be an excellent health tonic especially for a pregnant women and her child. No dosage	Singly	None	27
		Gall bladder	Dried	Diabetes	Swallowed whole. 1 whole piece once.	Singly	None	77
		Smoked dried hand	Dried	Mumps	It is used to tap gently in and around the affected area.	Singly	None	63
*Hystrix indica* (Kerr, 1792) /Hystricidae	Indian Crested Porcupine	Flesh	Dried or fresh	Easy delivery of a child	Cooked and eaten. No dosage	In combination with different vegetables	None	38
*Elephas maximus* (Linn,1758) /Elephantidae	Asian Elephant	Teeth	Dried or fresh	Toothache	The thick hair on the tail is used to pick the affected tooth.	Singly	None	4
*Lutrogale perspicillata* (Geoffroy, 1826) /Mustelidae	Smooth coated otter	Flesh	Dried or fresh	Fish bone stuck in the throat	Soup prepared is taken slowly. No dosage	Cooked with lady’s finger.	None	3
		Fur	Dried or fresh	Burns	Applied locally till it heals	Singly	None	2
*Trachypithecus pileatus* (Blyth, 1843) /Cercopithecidae	Capped langur	Tongue	Dried	Food poisoning	Cooked and eaten. No dosage	In combination with different vegetables	None	8
*Sus scrofa* (Linn. 1758) /Suidae	Wild Boar	Fat	Fresh	Hair care	Fats are fried to produce oil which is applied daily.	Singly	None	45
*Trachypithecus cristatus* (Raffles, 1821) /Cercopithecidae	Silvered Leaf Monkey	Gall bladder	Dried	Diabetes & High Blood Pressure	At least 3 pieces are eaten, one per day at intervals of a few days to lower diabetes. The same applies for high blood pressure.	Singly	None	82

**Table 2:** **Table showing the food value**, **other uses and recent conservation status of species mentioned in Table**1**as per the IUCN Redlist**

**Scientific Name/Family**	**English name**	**Vernacular name**	**Is the species Rare or Abundant?**	**Changes in abundance of the animal for the last 10 years (more Abundant/ Less Abundant/same/Rare)**	**Food value (Price in Indian Rupees if sold)**	**Uses other than food &medicine**	**Threat status**
							**(IUCN Red List)**
**Class: Reptilia**							
*Ophiophagus hannah* (Cant. 1836) /Elapidae	King Cobra	Sok ngan	Rare	Less Abundant	None	None	Vulnerable
*Python molurus* (Linn., 1758) /Pythonidae	Python	Rulpui	Rare	Rare	None	None	Near Threatened
*Varanus bengalensis* (Daudin, 1802) /Varanidae	Monitor lizard	Sartang	Rare	Rare	None	None	Least Concern
*Gekko gecko* (Linn. 1758) /Gekkonidae	Tokay Gecko	Totke	Abundant	Less Abundant	None (Rs. 2,50,000 approx for 300 gm live animal)	None	Not Assessed
*Melanochelys trijuga* (Schw.1812) /Geoemydidae	Indian Pond Terrapin	Sarpha	Rare	Rare	Yes (Rs 400 per kg)	Pet	Near Threatened
**Class: Malacostraca**							
*Paratelphusa* sp. (Alcok, 1919) /Paratelphisidae	Fresh water crab	Iai	Abundant	Less Abundant	Yes (Rs 5 per crab)	None	Not Assessed
**Class: Gastropoda**							
*Cryptozona* sp (Moerch, 1872) /Ariophantidae	Snail	Napkhong	Abundant	Same	None	None	Not Assessed
*Lymnaea* sp. (Lam. 1822) /Lymnaeidae	Water snail	Rifol	Abundant	Same	Yes (Rs 40 per kg)	None	Not Assessed
**Class: Insect**							
*Cimex lectularius* (Linn.1758) /Cimicidae	Bed bugs	Rifat	Abundant	Same	None	None	Not Assessed
*Periplanata americana* (Linn. 1758) /Blattidae	Cockroach	Khalai	Abundant	Same	None	None	Not Assessed
**Class: Aves**							
*Gallus gallus* (Linn. 1758) /Phasianidae	Jungle fowl	Ram Ar	Abundant	Less Abundant	Yes	Pet	Least Concern
*Corvus macrorhynchos* (Wagler, 1827) /Corvidae	Jungle crow	Va ak	Abundant	Same	None	None	Least Concern
*Rhyticeros undulatus* (Shaw, 1811) /Bucerotidae	Wreathed Hornbill	Rangkek	Rare	Rare	Yes	Used in traditional dance by the Zeme Naga Tribe	Not Assessed
*Passer domesticus* (Linn. 1758) /Passeridae	House Sparrow	Vasak	Abundant	Same	Yes	None	Least Concern
*Myophonus caeruleus* (Scopoli, 1786) /Turdidae	Blue whistling Thrush	Vakok	Rare	Rare	Yes	Pet	Least Concern
*Buceros bicornis* (Linn. 1758) /Bucerotidae	Great Hornbill	Vapual	Rare	Rare	Yes	Skull used as decoration	Near Threatened
*Upupa epops* (Linn. 1758) /Upupidae	Hoopoe	Vathitok/Vakhawri	Rare	Rare	Yes (Rs 1500 whole)	Feather used as decoration	Least Concern
**Class** : **Mammalia**							
*Manis crassicaudata* (E. Geof. 1803) /Manidae	Indian Pangolin	Saphu	Rare	Rare	Yes (Rs 10,000 whole skin)	The skin is believed to posses powers to ward off black magic.	Near Threatened
*Canis aureus* (Linn. 1758) /Canidae	Golden Jackal	Sihal	Abundant	Less Abundant	Yes (Rs 250 per kg)	None	Least Concern
*Muntiacus muntjak* (Zimm. 1780) /Cervidae	Barking Deer	Sakhi	Rare	Rare	Yes (Rs 400 per kg)	None	Least Concern
*Eonycteris spelaea* (Dobson, 1871) /Pteropodidae	Bat	Bak	Abundant	same	None	None	Least Concern
*Melursus ursinus* (Shaw, 1791) /Ursidae	Sloth Bear	Ivom	Rare	Rare	None	Pet	Vulnerable
*Ursus thibetanus* (Cuvier, 1823) /Ursidae	Himalayan Black Bear	Ivom	Rare	Rare	None	Pet	Vulnerable
*Hoolock hoolock* (Harlan, 1834) /Hylobatidae	Hoolock Gibbon	Saha	Rare	Rare (almost extinct)	Yes (Rs 250–300 per kg)	Pet	Endangered
*Capricornis sumatraensis* (Bechstein, 1799) /Bovidae	Mainland Serow	Sarza	Rare	Rare (almost extinct)	Yes (Rs 300 per kg)	Skin is used for making drums	Vulnerable
*Nycticebus coucang* (Boddaert, 1785) /Lorisidae	Slow Loris	Sahuai	Rare	Rare	Yes	As Pet	Vulnerable
*Canis lupus familiaris* (Linn. 1758) /Canidae	Dog	Ui	Abundant	Same	Yes (Rs 200 per kg)	Pet	Not Assessed
*Macaca assamensis* (McClelland, 1840) /Cercopithecidae	The Assamese Macaque	Zong	Abundant	Less Abundant	Yes (Rs 200–250 per kg)	Pet	Near Threatened
*Hystrix indica* (Kerr, 1792) /Hystricidae	Indian Crested Porcupine	Sarku	Rare	Rare	Yes (Rs 400–600 whole)	Spine used in traditional weaving	Least Concern
*Elephas maximus* (Linn,1758) /Elephantidae	Asian Elephant	Saipui	Rare	Rare (almost extinct)	Yes (Rs 300–500 per kg)	None	Endangered
*Lutrogale perspicillata* (Geoffroy, 1826) /Mustelidae	Smooth coated otter	Saram	Rare	Rare	Yes	None	Vulnerable
*Trachypithecus pileatus* (Blyth, 1843) /Cercopithecidae	Capped langur	Idor	Rare	Rare	Yes (Rs 250–300 per kg)	None	Vulnerable
*Sus scrofa* (Linn. 1758) /Suidae	Wild Boar	Sangal	Abundant	Same	Yes (Rs 250–300 per kg)	None	Least Concern
*Trachypithecus cristatus* (Raffles, 1821) /Cercopithecidae	Silvered Leaf Monkey	Ngau	Rare	Rare	Yes (Rs 200–250 per kg)	Pet	Near Threatened

**Table 3:** Ethnomedicinal uses of animals reported from different parts of India

**Title**	**No of species**	**Authors**	**Reference**
Sporadic study in India	20	Gosh A K, Maiti P K	[44]
Bhil of Rajasthan	17	Sharma S K	[43]
Bhil, Gamit, Kokna etc. of Maharastra	15	Patil S H	[42]
Chhattisgarh	10	Oudhia P	[41]
Chhattisgarh	7	Oudhia P	[40]
Kachch (Gujrat)	34	Gupta Leena et al.	[39]
Irular, Kurimba of Tamilnadu	26	Solvan A et al.	[38]
Kanikar, Paliyar of Taminadu	11	Ranjit Singh ASA	[37]
Mogya, Meena, Bawaria of Rajasthan	15	Mahawar, Jaroli	[33]
Saharia of Rajasthan	15	Mahawar, Jaroli	[36]
Monpas	11	Solanki GS and Chutia P	[11]
Mizoram	56	Lalramnghinglova	[12]
Chakhesang of Nagaland	23	Kakati and Doulo	[16]
Naga tribe of Nagaland	26	Jamir N S et al.	[14]
Dibrugarh (Assam)	4	Dilip Kalita	[15]
Ao tribe of Nagaland	25	Kakati L N et al.	[17]
Nyishi & Galo Tribe of Arunachal Pradesh	36	Chakravorty et al.	[13]
**Biate Tribe, Dima Hasao, Assam**	**34**	**Present Study**	

**Figure 4:**
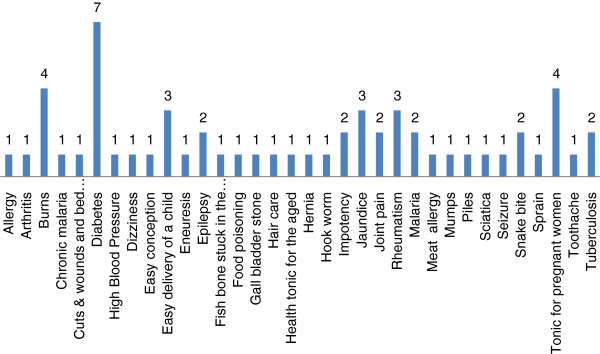
Number of species used by the Biate tribe against different diseases.

Fidelity Level (FL) demonstrates the percentage of respondents claiming the use of a certain animal for the same major purpose. The uses of animals that are commonly known by the respondents have higher fidelity level than less common known. The bone of *Hoolock hoolock* used to relieve rheumatism has the highest FL (97%) followed by *Paratelphusa sp* for the treatment of jaundice (96%) and the gall bladder of *Melursus ursinus* used for easy delivery of a child (93%). The use of the fur of *Lutrogale perspicillata* to treat burns and *Periplaneta americana* for tuberculosis has the lowest FL of 2% each (Table 1). The FL reveals that the treatment for frequently reported ailments has the highest FL value and those with low number of reports have low FL value. About 34 different ailments have been reported to be treated in the present study (Figure 4). While some of the ailments reported here are common ones such as burns, worms, hair care etc., but most of them are serious ailments such as jaundice, diabetes, malaria, impotency, hernia, epilepsy etc. Maximum number of species has been reported for the treatment of diabetes (Figure 4). The high fidelity levels for the treatment of diabetes suggest high frequency of report and thereby warrant in-depth studies to establish its pharmacological activity.

**Figure 5:**
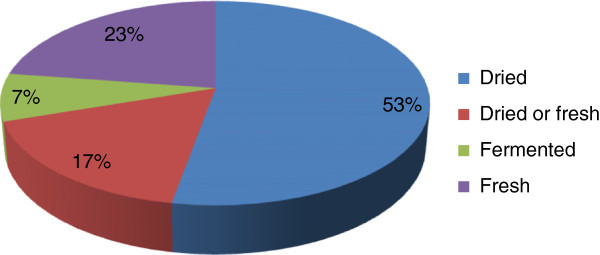
Proportional representation of species used in different forms.

**Figure 6:**
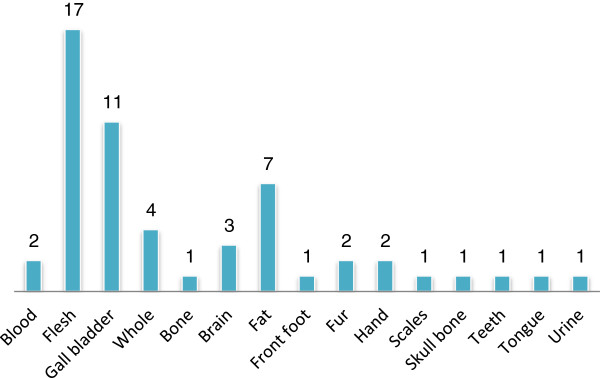
Animal parts used for medicinal purposes.

In several cases, medicine is prepared singly or in combination with herbs and very often cooked with vegetables to enhance the taste. While some are prepared fresh, some are prepared after it is sun dried or smoked dried. Fifty three percent of the animal parts are used after drying either after sun dried or smoked dried (Figure 5). Different parts of the animals are utilized for different ailments. In some cases, the sought after body parts does not always come from the same species. For example, the gall bladder from six different species has been reported as treatment for diabetes (Table 1). Maximum number of animal parts used for medicine comes from the flesh, followed by the gall bladder and fat (Figure 6). Very few side effects have been reported except for a few species.

The study has revealed that the Biate tribe possesses vast knowledge on the traditional usage of animal parts for medicine. This traditional knowledge is a guarded secret passed on from generation to generation through oral tradition. The reluctance of the traditional healers to reveal their secrets is due to the belief that revealing the properties and secrets renders the medicine ineffective. Although some of the species has already been documented elsewhere in India, the usages documented herein are unique to the Biate tribe. As for example, fats of *Buceros bicornis* is used by the Nyishi and Galo tribe of Arunachal Pradesh to treat body pain [13], while in the present study area, the fats of the same species is used in the treatment of arthritis. Flesh and fats of *Hystrix sp* are used in the treatment of body ache, rheumatic pain etc. [14] however the flesh of the same species is used to facilitate easy delivery of a child amongst the Biate tribe. Tooth of *Elephas maximus* is used for conjunctivitis and pimples [38], whereas it is used for toothache by the Biate tribe. Similarly, the use of *Python molurus* flesh for seizure, *Cimex lectularius* for malaria, flesh of *Upupa epops* for gall bladder stone etc. are all unique to the Biate tribe.

It has been observed that most of the animal parts are usually procured manually which involves hunting of these animals and birds. Very often, animal-derived medicines and preserved animal body parts are sold openly at the local markets. Commercialization of animals for medicinal purposes is a widespread phenomenon; with significant implications for their conservation and sustainable use [45]. Local villagers very often hunt these animals in the lure of quick money. For example, a 300 gm live *Gekko gecko* may fetch a sum of 2,50,000 INR and scales of *Manis crassicaudata* cost approximately 10,000 INR (Table 2). Thus, the unchecked and unmonitored sale of wild animal products in the local markets has a significant impact on the population of the wild fauna.

**Figure 7:**
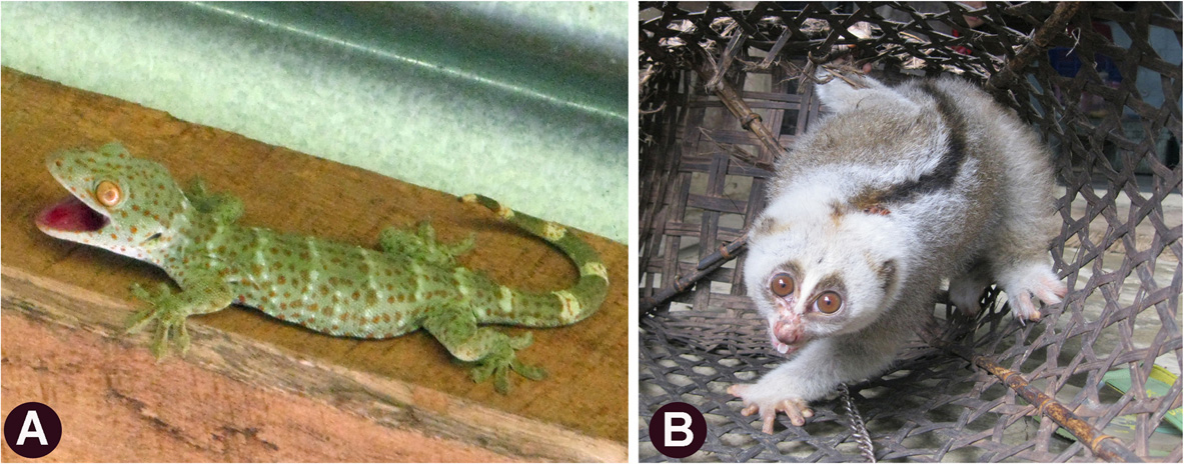
**Two of the most sought after species of Dima Hasao, A. *Gekko gecko *Linn. B. *Nycticebus coucang *Boddaert.**

The present study has documented the usage of at least 15 animals listed under Endangered, Vulnerable and Near Threatened category of the IUCN Red List (Table 2). While several are listed under Least Concern in the IUCN Red List, the present study observes that there has been a drastic change in the abundance of the species even within the last ten years (Table 2). Species like *Gekko gecko*, *Gallus gallus*, *Canis aureus*, *Macaca assamensis*, *Nycticebus coucang* are less abundant now as compared to the last ten years (Figure 7).

The present study has revealed that out of the 34 species documented herein, at least 24 are also hunted for its meat as much as it is hunted for its medicinal value. Smoked meat of *Sus scrofa*, *Trachypithecus pileatus*, *Hoolock hoolock* etc. are in high demand and cost approximately 250–300 INR per kg (Table 2). Wild edible plants and animals form a part of the basic source of food amongst the tribal community in the study area. Traditionally, most of the Biate villages have an age old system of sustainable utilization which involves protecting or closing a section of the village forest area for several years and then open it for several years while another section is protected. The village chief is the supreme head and owner of the entire land and it is he who ensures the protection of an area along with his council. However, presently, with the lands coming under the Autonomous District Council, the village chiefs are often appointed by the district council and doesn’t follow the clan system as earlier and although the village chief is still the head, his powers are limited and the sense of ownership is gradually declining leading to unimpeded exploitation of the forest resources by the villagers and also by outsiders. *Elephas maximus*, *Capricornis sumatraensis* and *Hoolock hoolock* has almost been obliterated in the study area due to its excessive hunting. This is a fact of great concern because those species directly involved in traditional medicines and food should be amongst those of the highest priority for conservation [46].

Besides its medicinal and food value, these animals are also hunted for other uses. In a Biate community, as in most of the tribes of the Chin-Kuki-Mizo clan of the district, the traditional drum known as ‘*Khuang*’ plays an important role while performing rites and rituals in the early days. Even in the present day, after the advent of Christianity, the ‘*Khuang*’ is still an integral part of the church music. The hide of *Capricornis sumatraensis* is considered one of the best raw materials for making the ‘*Khuang*’,, and almost every household has one. The unrestricted hunting of this particular species for its meat and hide has almost wiped out the population within the district. Species like, *Melanochelys trijuga*, *Macaca assamensis*, *Nycticebus coucang*, *Hoolock hoolock* etc. are also reared as pets while skull and feather of *Buceros bicornis*, *Upupa epops* etc. are used as decoration and as a sign of expertise in hunting. The spine of *Hystrix indica* is used in weaving (Table 2).

Thus, it is important to point out that although medicinal use of animals is one of the main cause of threats to wild populations, it cannot be considered the only threat to the conservation of the species [47]. Medicinal use of animals must be considered together with other anthropogenic pressures, such as habitat loss, hunting for food etc. In most cases, the medicinal products of animals are by products from animals hunted for other purposes; thus, these multiple uses (including medicinal) of fauna and their impact on animal populations must be properly assessed and taken into consideration when implementing recovery plans for these species, especially those that are highly exploited [48].

It was observed that most of the people prefer this traditional cure to the modern pharmaceuticals, as it is less expensive and claimed to be more effective. Thus, zootherapy is an important and integral part of the traditional healthcare system of a tribal community, however overexploitation and lack of regulation and monitoring to safeguard for sustainable utilization is a point to consider seriously from the conservation point of view. Active participation by stakeholders at various levels in any management strategies, especially youths and the younger generation could help guarantee the sustainable use of these zootherapeutic resources. Spreading awareness coupled with a holistic approach to *in situ* and *ex situ* conservation, clinical trials and multidisciplinary studies to establish the efficacy of the products could come a long way in safeguarding the traditional knowledge and the resources as well.

## Conclusion

The study provides a comprehensive account of the vast wealth of traditional knowledge and healthcare system of Biate tribe inhabiting the Dima Hasao district of Assam. Careful scientific scrutiny and screening of this traditional knowledge could lead to the development of newer and safer drugs as well as spur conservation and sustainable utilization of such a unique habitat and resources. The study area warrants further studies to document the traditional knowledge of the rest of the tribes inhabiting the area, which is the most important aspect in determining practical approaches to the management and sustainable use of the local flora and fauna.

## Competing interest

The author(s) declare that they have no competing interest.
